# A TaqMan-Based Multiplex qPCR Assay and DNA Extraction Method for Phylotype IIB Sequevars 1&2 (Select Agent) Strains of *Ralstonia solanacearum*

**DOI:** 10.1371/journal.pone.0139637

**Published:** 2015-10-01

**Authors:** Michael J. Stulberg, Qi Huang

**Affiliations:** 1 Floral and Nursery Plants Research Unit, Agricultural Research Service, United States Department of Agriculture, Beltsville, Maryland, United States of America; 2 Oak Ridge Institute for Science and Education, Oak Ridge, Tennessee, United States of America; Dong-A University, REPUBLIC OF KOREA

## Abstract

*Ralstonia solanacearum* race 3 biovar 2 strains belonging to phylotype IIB, sequevars 1 and 2 (IIB-1&2) cause brown rot of potato in temperate climates, and are quarantined pathogens in Canada and Europe. Since these strains are not established in the U.S. and because of their potential risk to the potato industry, the U.S. government has listed them as select agents. Cultivated geraniums are also a host and have the potential to spread the pathogen through trade, and its extracts strongly inhibits DNA-based detection methods. We designed four primer and probe sets for an improved qPCR method that targets stable regions of DNA. RsSA1 and RsSA2 recognize IIB-1&2 strains, RsII recognizes the current phylotype II (the newly proposed *R*. *solanacearum* species) strains (and a non-plant associated *R*. *mannitolilytica*), and Cox1 recognizes eight plant species including major hosts of *R*. *solanacearum* such as potato, tomato and cultivated geranium as an internal plant control. We multiplexed the RsSA2 with the RsII and Cox1 sets to provide two layers of detection of a positive IIB-1&2 sample, and to validate plant extracts and qPCR reactions. The TaqMan-based uniplex and multiplex qPCR assays correctly identified 34 IIB-1&2 and 52 phylotype II strains out of 90 *R*. *solanacearum* species complex strains. Additionally, the multiplex qPCR assay was validated successfully using 169 artificially inoculated symptomatic and asymptomatic plant samples from multiple plant hosts including geranium. Furthermore, we developed an extraction buffer that allowed for a quick and easy DNA extraction from infected plants including geranium for detection of *R*. *solanacearum* by qPCR. Our multiplex qPCR assay, especially when coupled with the quick extraction buffer method, allows for quick, easy and reliable detection and differentiation of the IIB-1&2 strains of *R*. *solanacearum*.

## Introduction

Bacterial wilt caused by *Ralstonia solanacearum* (*Rs*) is a xylem disease causing vascular dysfunction in susceptible hosts such as zonal geranium (*Pelargonium* x *hortorum*) (geranium), tomato and potato. *Rs*, a soil-borne pathogen that is difficult to eradicate from endemic areas [1], is a species complex that consists of strains with different host ranges and biochemical properties which are the basis for two traditional classification schemes: race and biovar [2]. Typically, race and biovar are not correlative, except that biovar 2 strains are almost always race 3, resulting in the classification race 3 biovar 2 (r3b2). Thought to originate from the Andes Mountains, r3b2 strains are pathogenic mainly to potato, tomato, and geranium, as well as solanaceous weeds, and they are well adapted to cool-temperate climates [3,4]. This group of *Rs* strains is listed as select agents by the U.S. government [5] because of their potential threat to U. S. agriculture. The terms race and biovar are used by the federal government to define select agent strains of *Rs*.

Over the last fifteen years, genomic sequencing and phylogenetic analyses have increased our knowledge and understanding of the relationships among strains in the *Rs* species complex, leading to a new classification scheme. A phylotyping method based on 16S-23S intergenic linker sequence data grouped all *Rs* species complex strains into four phylotypes that correspond to their geographic origins: Asia (phylotype I), the Americas (phylotype II), Africa (phylotype III), and Indonesia (phylotype IV, which includes *R*. *syzygii* and the blood diease bacterium (BDB)) [6]. Further phylogenetic analysis based on the *endoglucanase* gene sequence grouped strains into sequevars, with the previously known r3b2 strains being in the phylotype IIB, sequevar 1 and 2 clusters (IIB1&2) [6]. This advancement in genetic comparisons and analysis has allowed for improved DNA-based diagnostics. Recently, a proposal has been made to split the *Rs* species complex into three different *Ralstonia* species [7]. Under the proposed change, only the current phylotype II strains would continue to be *Rs*, while phylotype I and III strains would be *R*. *pseudosolanacearum* and phylotype IV strains would be *R*. *syzygii* with multiple subspecies. Although a multiplex PCR assay is available to distinguish the four phylotypes of *Rs* [6], no progress has been made to develop a qPCR assay that amplifies only the newly proposed *Rs* strains (phylotype II).

One disease control strategy for *Rs* involves using propagative plant tissue that is free of the pathogen. Pathogen-free tissue can be verified by rapid and sensitive screening tests such as TaqMan-based quantitative real-time PCR (qPCR). Screening imported geranium cuttings for quarantined pathogens is also an important preventative and exclusion measure, as geraniums have been a major source of accidental introductions of *Rs* r3b2 into the US, Canada, and Europe [8–11]. Over the past 20 years, many PCR-based methods to detect *Rs* at the species level have been published, including several assays developed to amplify ribosomal rRNA sequences (i.e. 16S or 16S-23S intergenic spacer region), as well as more recent efforts to detect genes such as *endoglucanase*, *hrpB*, *hrpu*, *flic*, cytochrome *c*1 signal peptide, the upstream region of UDP-3-*O*-acyl-GlcNAc deacetylase, and a predicted glycosyl transferase [12–19]. For detection at the biovar 2/IIB-1&2 level, only four conventional PCR assays [20–23], two loop-mediated isothermal amplification (LAMP) assays [22,24] and two qPCR assays [13,25] have been developed so far.

Fegan and Prior developed the first biovar 2 specific PCR primer pair 630/631 based on Southern hybridization and competitive hybridization [21]. Although extensively used over the years, this primer set has produced false-positive reactions from strains isolated in South America [21,23] and targets a potential phage-related mobile element [20]. *In silico* genomic comparisons to identify r3b2/IIB-1&2-specific DNA for detection yielded promising results, but the specific primer pairs designed for PCR were either not fully tested [20] or targeted a potential mobile element [22]. We recently developed a multiplex PCR assay that targets non-phage genome sequences and allows for simultaneous detection and differentiation of *Rs* species complex and IIB-1&2 strains with an internal plant control [23].

For qPCR, the most widely used biovar 2 specific primers and probe set can be used in a duplex qPCR assay to simultaneously detect biovar 2 strains and either *Rs* species complex strains or an internal control, but not all three [13,26]. This set was designed based on the same, although shortened, region of the 630/631 primer pair, and was found even more susceptible to false positives [27]. A SYBR Green qPCR assay developed for r3b2/IIB-1&2 detects strain UW349, a IIB-27 strain of *Rs* [23,25]. Recently, a multiplex qPCR assay was developed to detect race 3 strains of *Rs*, but this assay also detects non-IIB-1&2 biovar 1 strains of *Rs* [28]. The most recent LAMP assay developed to detect r3b2/IIB-1&2 strains is specific and is duplexed to also detect the *Rs* species complex strains, but does not include an internal plant control to exclude false-negatives [24].

Imported geranium cuttings have been accidental carriers of r3b2 into the U. S. and some European countries [8,11], and components in extracts of this plant species strongly inhibit DNA-based amplification methods [23,25]. Detection of *Rs* in geranium cuttings requires DNA extraction methods that negate PCR inhibitors and internal reaction controls to validate the process. Primers designed to amplify plant DNA as a qPCR internal reaction control were designed for *Solanaceous* species and do not recognize geranium [13,28]. An internal reaction control plasmid has been used with qPCR detection of biovar 2, but it does not control for a successful plant DNA extraction [26]. We have recently designed internal control primers that allow detection of plant DNA by PCR from 8 tested plant species including tomato, tobacco, potato and geranium [23], but the amplicon is too large for qPCR analysis. DNA extraction methods designed to negate PCR inhibition are available either commercially or developed by researchers, but they are expensive, time-consuming, require specialized materials, or have varying success [13,25,29,30].

Our goals were not only to improve the specificity of IIB-1&2 strain detection by qPCR, but also to develop a multiplex qPCR assay that targets the newly proposed *Rs* species strains (phylotype II) as a secondary confirmation of IIB-1&2 identification. We also included internal plant DNA control primers and probes that recognized all major hosts of *Rs* including tomato, potato, tobacco and geranium, in order to validate plant extractions and qPCR reactions. Such multiplex qPCR assays are urgently needed in light of the recent changes to the select agent regulations that list all *Rs* species complex strains as select agents until further testing can differentiate them from r3b2 strains [31]. We also developed a simple extraction method using an extraction buffer we designed to reduce PCR inhibition from geranium tissues foreasy and fast processing of infected plant samples.

## Materials and Methods

### Bacterial strains and DNA extraction

The same ninety *Rs* species complex strains we used previously [23] were used in this study, including 34 r3b2 phylotype IIB seq 1 and 2 strains (select agent (SA)) and 56 non-SA strains of *Rs*. We also used 12 non-*Rs* strains, including phytobacterial species of *Enterobacter*, *Pseudomonas*, and *Xanthomonas*, as well as four other non-plant associated but closely related *Ralstonia* species to *Rs* for a thorough test (Table 1).

**Table 1:** Bacterial strains used in this study and tested by our primers and probes, under both uniplex and multiplex qPCR conditions.

Strain	Biovar; Phylotype-Sequevar^a^	TaqMan-based qPCR Cq value^b^						
		Uniplex qPCR				Multiplex qPCR		
		RsSA1	RsSA2	RSII	Cox1^c^	RsSA2	RSII	Cox1^d^
*Select agents (R3B2)*								
UW120 (S214)	2; IIB-1	21.85	22.12	21.09	-	22.97	21.41	-
UW150 (K0777)	2; IIB-1	21.81	22.36	19.51	-	22.55	20.04	-
UW220a	2; IIB-1	20.98	20.44	18.82	-	21.28	19.24	-
UW224	2; IIB-1	22.00	21.55	20.44	-	22.35	20.77	-
UW257	2; IIB-1	20.25	20.08	17.98	-	20.57	18.46	-
UW260	2; IIB-1	21.05	20.33	18.94	-	20.94	19.55	-
UW276	2; IIB-1	21.00	20.77	19.13	-	21.26	19.32	-
UW344 (10 1SC)	2; IIB-1	20.77	20.30	18.96	-	21.02	19.20	-
UW425 (O249)	2; IIB-1	20.98	20.76	18.84	-	20.95	19.19	-
UW449 (CIP259)	2; IIB-1	21.32	20.33	19.23	-	21.41	19.59	-
UW492 (CIP302)	2; IIB-1	20.39	20.37	18.55	-	20.37	19.03	-
UW501(CIP181)	2; IIB-1	21.39	21.09	20.37	-	21.48	20.20	-
UW551 (I-35)	2; IIB-1	21.30	20.76	20.19	-	22.63	20.85	-
UW552	2; IIB-1	19.83	19.34	17.17	-	19.77	17.73	-
Pss1370	2; IIB-1	22.29	21.62	20.32	-	22.48	20.80	-
Pss1475	2; IIB-1	21.42	20.99	19.75	-	21.49	20.01	-
Pss1586	2; IIB-1	20.69	20.64	19.03	-	21.13	19.39	-
JT516	2; IIB-1	21.82	21.56	19.83	-	22.00	20.27	-
CMR34 (CFBP7029)	2; IIB-1	21.45	20.81	19.31	-	21.44	19.68	-
PSS525	2; IIB-1	22.00	21.54	19.61	-	22.12	20.24	-
CMR24 (CFBP7027)	2; IIB-1	21.70	21.28	19.73	-	21.99	20.09	-
RE	2; IIB-1	21.59	21.11	19.45	-	21.71	19.76	-
IPO1609	2; IIB-1	22.16	21.58	19.79	-	22.17	20.22	-
IVIA1602.1	2; IIB-1	21.71	20.96	19.17	-	21.34	19.58	-
NCPPB4155	2; IIB-1	21.64	21.22	19.41	-	21.81	19.85	-
NCPPB4153	2; IIB-1	22.16	21.69	20.23	-	22.48	20.61	-
NCPPB2505	2; IIB-1	20.86	20.73	19.07	-	21.15	19.72	-
NCPPB1584	2; IIB-1	21.45	21.06	19.43	-	21.56	19.69	-
CSL Pr 3467	2; IIB-1	20.34	20.29	18.14	-	20.49	18.52	-
CSL Pr 3468	2; IIB-1	20.83	20.29	18.88	-	21.05	19.24	-
CSL Pr 1328	2; IIB-1	21.19	20.30	18.49	-	20.92	18.93	-
UW80 (CIP309)	2; IIB-2	21.31	20.36	19.08	-	20.73	19.25	-
CFBP3879	2T; IIB-2	21.68	21.23	19.88	-	21.85	20.20	-
CFBP1410	2T; IIB-2	22.18	21.83	20.92	-	22.25	21.30	-
*Non-select agents*								
UW349	2T; IIB-27	-	-	20.12	-	-	20.40	-
UW9 (S147)	1; II	-	-	17.95	-	-	18.23	-
K60	1; IIA-7	-	-	18.81	-	-	19.37	-
Rs5	1; II	-	-	19.45	-	-	19.88	-
Rs116	1; II	-	-	17.88	-	-	18.47	-
Rs124	1; II	-	-	20.33	-	-	20.74	-
Rs126	1; II	-	-	18.81	-	-	18.85	-
Rs129	1; II	-	-	19.81	-	-	19.86	-
IBSBF1503	1; IIB-4NPB	-	-	19.94	-	-	20.12	-
LNPV24.25	1; IIB-4NPB	-	-	19.31	-	-	19.80	-
P446	1; IIB-4	-	-	18.41	-	-	18.93	-
P487	1; IIB-4	-	-	19.13	-	-	19.16	-
P506	1; IIB-4	-	-	19.49	-	-	20.21	-
P673	1; IIB-4	-	-	21.99	-	-	22.33	-
P618	1; IIB-4	-	-	20.97	-	-	21.42	-
P597	1; IIA-37	-	-	21.31	-	-	21.32	-
P550	1; IIA-7	-	-	20.79	-	-	21.09	-
Molk2	1; IIB-3	-	-	20.09	-	-	20.48	-
UW119 (S213)	3; I	-	-	-	-	-	-	-
Rs121	3; I-13*	-	-	-	-	-	-	-
Pss32	3; I	-	-	-	-	-	-	-
Pss530	3; I	-	-	-	-	-	-	-
Pss4	3; I-15	-	-	-	-	-	-	-
Pss266	3; I	-	-	-	-	-	-	-
Pss97	3; I-34*	-	-	-	-	-	-	-
Pss185	3; I	-	-	-	-	-	-	-
Pss201	3; I	-	-	-	-	-	-	-
Pss278	3; I	-	-	-	-	-	-	-
Pss221	3; I	-	-	-	-	-	-	-
Pss106	3; I-34*	-	-	-	-	-	-	-
Pss73	3; I	-	-	-	-	-	-	-
Pss18	3; I	-	-	-	-	-	-	-
Pss71	3; I-34*	-	-	-	-	-	-	-
HB512	3; I	-	-	-	-	-	-	-
JS526	3; I	-	-	-	-	-	-	-
GZ519	3; I-17*	-	-	-	-	-	-	-
FJ47	3; I	-	-	-	-	-	-	-
GX53	3; I-44*	-	-	-	-	-	-	-
JS526	3; I	-	-	-	-	-	-	-
GMI1000	3; I-18	-	-	-	-	-	-	-
UW151	4; I-16*	-	-	-	-	-	-	-
Pss191	4; I-15*	-	-	-	-	-	-	-
Pss565	4; I	-	-	-	-	-	-	-
Pss901	4; I	-	-	-	-	-	-	-
Pss51	4; I-15*	-	-	-	-	-	-	-
Pss228	4; I	-	-	-	-	-	-	-
Pss114	4; I	-	-	-	-	-	-	-
Pss267	4; I	-	-	-	-	-	-	-
Pss262	4; I	-	-	-	-	-	-	-
Pss1655	4; I	-	-	-	-	-	-	-
Pss1283	4; I	-	-	-	-	-	-	-
JT525	1; III-19	-	-	-	-	-	-	-
CMR15 (CFBP6941)	2T; III-29	-	-	-	-	-	-	-
PSI07	2T; IV-10	-	-	-	-	-	-	-
BDB R229	IV-10	-	-	-	-	-	-	-
*R*. *syzygii* R24	IV-9	-	-	-	-	-	-	-
*non-R*. *solanacearum*								
*R*. *pickettii* ATCC27511		-	-	38.20	-	-	34.71	-
*R*. *insidiosa* ATCC49129		-	-	-	-	-	37.00	-
*R*. *mannitolilytica* ATCC BAA-716		-	-	20.79	-	-	22.51	-
*Cupriavidus necator* ATCC17699		-	-	-	-	-	-	-
*Enterobacter cloacae*		-	-	-	-	-	-	-
*Xanthomonas campestris pv*. *campestris strain 6*						-	-	-
*X*. *campestris pv*. *campestris strain 7*		-	-	-	-	-	-	-
*X*. *campestris pv*. *campestris strain 8*						-	-	-
*X*. *campestris pv*. *pelargonium strain 24*						-	-	-
*X*. *campestris pv*. *pelargonium strain 25*						-	-	-
*Psuedomonas syringae pv*. *syringae*		-	-	-	-	-	-	-
*X*. *citri*		-	-	-	-	-	-	-

^a^Sequevars with

* were determined in this study.

^b^Mean Cq values of two replicated experiments.

^c^Both Cox1 sets with probe Cox1-P-S or Cox1-P-P were tested separately in a uniplex qPCR and yielded the same result.

^d^The Cox1 duplexed probe set containing Cox-1-qF/P/P-S/P-P were used in the multiplex qPCR.

*Rs* was grown, its inocula prepared, and final inoculum cell concentration confirmed as described previously [23]. Genomic DNA was extracted from the 99 bacterial strains in Table 1 using the Blood and Tissue kit (Qiagen, Valencia, CA) according to the manufacturer’s instructions.

### Sequevar analysis

The endoglucanase (*egl*) gene fragments of GX53, GZ519, Pss106, Rs121, and UW151 were amplified and sequenced as described before [23]. The untrimmed, 2x coverage *egl* sequences were submitted to GenBank and given accession numbers KT355482, KT355483, KT355484, KT355485, and KT355486. The *egl* sequences of unknown sequevars Pss71 (EU407269), Pss97 (EU407272), Pss51 (EU407266), and Pss191 (EU407286) were downloaded from GenBank. The sequevars of the 9 strains were determined by building a phylogenetic tree with *R*. *solanacearum egl* sequences representing almost all of the sequevars as previously described [23].

### Plant growth, inoculation, and DNA extraction

Seeds of tomato (*Lycopersicom esculentum Mill*. *cv*. ‘bonnie best’) and geranium (*Pelargonium x hortorum* ‘zonal geranium’) were grown as described previously [23]. Seedlings were inoculated two to seven days after transplanting, using root wounding followed by a soil-drenching inoculation method. The roots were wounded by cutting across each pot through the soil 1-cm from the base of the stem with a sterile scapel. Afterwards, 40 mL of *Rs* cell suspension at a concentration of 2 x 10^7^ CFU/ml were poured into each pot for each tested strain. Plants were inoculated either with a IIB-1 strain UW551 or UW344, or with a non-IIB-1&2 strain K60 or Rs5; all four are phylotype II strains of *Rs*. Water was used as a negative control. To obtain asymptomatic plant samples, a total of 32 tomato stem samples were taken with nine collected at two, three, and four days, respectively, and five at five days post inoculation (dpi). A total of 54 asymptomatic geranium samples were collected with 12 stem samples taken each day from 3 to 6 dpi, and 6 within 3 weeks of being infected and not displaying symptoms. The asymptomatic plant samples were collected by cutting the stem 1 cm above the soil line, removing the lowest 1 cm stem after surface sterilization by wiping with a paper towel soaked with 70% ethanol for 30 sec, and cutting two 2- or 5-mm sections from the remaining geranium or tomato stem, respectively. One section was used for DNA extraction and the other for bacterial isolation as described below. To obtain symptomatic plant samples, stems from four tomato and 18 geranium were collected the same way as described for the asymptomatic plant samples after they displayed varying degrees of wilt symptoms and their DNA was also extracted. One stem from a water-inoculated geranium plant was also collected for DNA extraction. DNA extracted previously from healthy and *Rs*-infected symptomatic tomato (25), geranium (4), tobacco (*Nicotiana tabacum*) (5) and potato (*Solanum tuberosum*) (3), as well as from healthy oleander (*Nerium oleander*), vinca (*Catharanthus roseus*), begonia (*Begonia semperflorens-cultorum*) and impatiens (*Impatiens walleriana*) were also used for this study [23]. DNA was also extracted from 23 additional potato plants grown, inoculated and sampled as described before [23].

### Bacterial isolation from asymptomatic tomato and geranium plants

To determine bacterial concentration in asymptomatic plant samples, plant stem sections described above were put into 500 μL sterile water in a 1.7 mL micro-centrifuge tube. The tissue was ground with a disposable pestle and dilution plated [23]. Bacterial concentration was determined by averaging two plates per dilution per plant sample with countable bacterial cells.

### Primer and probe design

Primers were designed using the free online A plasmid Editor (ApE) program with specific design parameters (GC = 45 to 60%, Tm = 60 to 64°C, primer length 18 to 26 bp), and probes were designed using the PrimerQuest tool on the Integrated DNA Technologies’ (IDT, Coralville, IA) website. RsII primers and probe were designed targeting the 16S-23S intergenic sequence that was used previously in the multiplex PCR assay for phylotype determination of *Rs* strains [6]. For the plant internal control, the region of the cytochrome oxidase subunit 1 (*cox1*) gene we used previously [23] was shortened to a length amenable to qPCR. Two probes were designed to the *cox1* region; one to recognize solanaceous plants and the other to recognize pelargonium. The solanaceous plant probe Cox1-P-S was designed using sequence from *Solanum tuberosum* mitochondrial cox1 gene (GenBank X83206) and the pelargonium probe Cox1-P-P was designed using sequence from the *Pelargonium x hortorum* cox1 gene (GenBank DQ317047). Probes were synthesized by IDT using 6-FAM, TET, and Tex615 fluorescent reporter dyes at the 5’ end of specific probes for RsSA, RsII and Cox1, respectively. Single quenched probes were constructed using Iowa Black FQ and Iowa Black RQ Sp at the 3’ end of RsSA2 and Cox1 probes, respectively (Table 2). Double quenched probes using an internal ZEN quencher nine base pairs from the 5’ end and Iowa Black FQ at the 3’ end were constructed for RsSA1 and RsII probes.

**Table 2:** Primers and probes used in this study.

Set		Sequences (5’– 3 ‘)	Target	Amplicon (bp)	Specificity^a^
	F^b^	CAACGATGCCTGGAAACTGACC	Predicted		
RsSA1	R^b^	TGGTCCGGGTTCAGGTAAATGTCAC	ferric siderophore	132	All IIB-1&2 strains of *Rs*
	P	FAM/CCCGACATC/Zen/TACAACATCAGCACCAACG/IBFQ	receptor		
	F	TGCTGAATTCGTTCGGGTGATG	Probable n-		
RsSA2	R	CGTTCCAAGTAGTGGGCAATCAA	6 adenine-specific	93	All IIB-1&2 strains of *Rs*
	P	FAM/CCAAGAAGAGAATCATGGAGCCGTTGTCC/IBFQ	DNA methylase		
	F	GTTATGGACGGTGGAAGTCTCTG	16S-23S		All *Rs*
RsII	R	CACAAGGTATTGGTGGAGGATGA	ITS region	117	phylotype II
	P	TET/TCAGCTGGG/Zen/AGAGCACCTGCTTTG/IBFQ			strains and *R*. *mannitolilytica*
	qF	GGTGTTCTTGGATTTCTTGTTTGGGC			
Cox1	R^b^	CCACATGGTAGCGATCCAACTAAAGAT			All plant
	P-S	TEX615/AGCCTACTTCACCGCAGCTACCATGATCATAGC/IBRQ	*cox1*	144	species
	P-P	TEX615/ACGAGAGCCTACTTTACTGCAGCTACT/IBRQ			

^a^Primers and probes detected only the tested *Rs* species complex strains and tested plant species including geranium (*Pelargonium x hortorum*), potato, tomato, tobacco, impatiens, salvia, vinca, and oleander.

^b^From Stulberg et al. (2015) [23].

### qPCR conditions

qPCR assays were performed in a 20 μl volume containing either iQ Supermix for uniplex or iQ Multiplex Powermix (Bio-Rad Laboratories, Hercules, CA) for multiplex reactions. Approximately 3 ng of bacterial genomic DNA or 20 ng of DNA extracted from healthy or *Rs*-infected plant samples was added to the qPCR reactions. A final concentration of 2 μM of each primer and 1 μM of each probe was used in both uniplex and multiplex reactions. Cycling conditions for uniplex reactions with RsSA1-F/R/P, RsSA2-F/R/P, or Cox1-qF/R/P-S/P-P were 95°C for 3 minutes, 40 cycles of 95°C for 10 seconds and 62°C for 30 seconds. For the RsII-F/R/P uniplex reaction and the multiplex qPCR assay containing three primer pairs (RsSA2-F/R, RsII-F/R and Cox1-aF/R) and four probes (RsSA2-P, RsII-P, Cox1-P-S and Cox1-P-P), cycling parameters were 95°C for 3 minutes, 40 cycles of 95°C for 10 seconds and 66°C for 30 seconds. Weller et al.’s (2000) cox1 primer and probe set was tested according to their protocol [13]. All qPCR assays were performed in a CFX96 thermo-cycler (Bio-Rad Laboratories, Hercules, CA).

### Standard curves

Primer and probe efficiency and the limit of detection in both multiplex and uniplex qPCR were performed in BioRad’s iQ Multiplex Powermix containing cells from *Rs* strain UW551. Template cells were diluted to a starting concentration of 2 x 10^7^ CFU/mL and then serially diluted by 10-fold increments. Dilutions were made either in sterile water or in 20 ng of Qiagen-extracted total plant DNA from healthy geranium. All reactions were performed in duplicate and the experiment was run two times. Water was used as a no-template control.

### Comparison of extraction methods

Different concentrations and combinations of Tris-HCl, NaCl, Betaine monohydrate, polyvinyl-pyrrolidone (PVP), and Triton X-100 at different pH (all chemicals were from Sigma-Aldrich, St. Louis, MO) were tested to develop an effective extraction buffer. Total plant DNA extractions were performed with four different methods, side by side, on symptomatic tomato and both symptomatic and asymptomatic geranium plants using 1) our extraction buffer (100 mM Tris-HCl, 150 mM NaCl, 1 M Betaine monohydrate, 10% polyvinyl-pyrrolidone, and 5% Triton X-100, pH 9.3), 2) Epicentre’s QuickExtract Seed DNA Extraction Solution (Epicentre, Madison, WI), 3) Qiagen’s DNeasy Plant Mini Kit (Qiagen, Valencia, CA), and 4) sterile water. The detection limit for each of the extraction methods was determined using healthy geranium plant extract spiked with 4 to 4 x 10^4^ CFU of UW551 per qPCR reaction. To extract DNA from symptomatic tomato plants, the stems were cut into 0.5 cm sections and added to each extraction buffer (Epicentre method requires less tissue, so a 2 mm section was used). To extract DNA from symptomatic and asymptomatic geranium plants, the stems were cut into two 2 mm sections and each section further divided into 4 parts, with two parts (approximately 50 to 150 mg) placed into each extraction buffer except that only 1 part was added to Epicentre’s. To use our extraction buffer for a quick extraction, the stem tissue was added to a 1.7 ml microcentrifuge tube containing 500 μl of our extraction buffer and incubated at 65°C with shaking at 1100 RPM in a MultiTherm Shaker (Benchmark Scientific, Edison, NJ) for 10 minutes. Samples were used immediately or stored at -20°C for up to 1 day. Sterile water was used in place of our buffer as a control. To use Epicentre’s method, 100 μl of buffer was used and the manufacturer’s instruction was followed. To use Qiagen’s method, DNA was eluted in 200 μl elution buffer. Each extraction was done using a total of 4 tomato and 30 geranium plants in two separate experiments. After all extractions, 1 μl from each method was used in a qPCR reaction.

### Statistical analysis

The qPCR results following extraction from four different techniques were analyzed for significant differences. Each primer and probe set was compared among the four different extraction techniques. Cq values were analyzed by one-way ANOVA using web-based statistical software (http://vassarstats.net/anova1u.html). Means were compared using the Tukey’s Honest Significant Difference test provided by the software.

## Results

### Design and test of primers and probes targeting phylotype II and IIB-1&2 strains of Rs, as well as plant DNA by uniplex qPCR

We designed two sets of primers and probe, RsSA1 and RsSA2, for the specific detection of SA/IIB-1&2 strains of *Rs* (Table 2). The RsSA1 primers were designed previously to amplify a 132 bp target. Since the amplicon size is suitable for a qPCR assay, we only designed the probe RsSA1-P for the RsSA1 set in this study (Table 2). The RsSA2 primers and probe were designed based on another non-phage related SA/IIB-1&2-specific sequence previously identified by our group [23], which also aligned closely to the RRSL1249 region identified by Kubota et al. [22], and the IPO_02103 sequence identified by Guidot et al. [20]. The primer pair RsSA2-F/R targets a 93-bp region that is part of an annotated adenine DNA methyltransferase-like protein in the UW551 draft genome and has 100% identity with nucleotides 5037 to 5129 in contig 0535 of UW551, and 2297674 to 2297766 in the draft genome of IPO1609 (GenBank LN651282). When tested by uniplex qPCR, both RsSA1 and RsSA2 sets detected the 34 IIB-1&2 strains, but none of the 56 non-IIB-1&2 strains of *Rs*, nor any of the 8 other plant-, soil-, or human-associated bacteria, including *E*. *cloacae*, *X*. *campestris pv*. *campestris*, *P*. *syringae pv*. *syringae*, *X*. *citri*, *Cupriavidus necator* (formerly *R*. *eutropha*), *R*. *pickettii*, *R*. *insidiosa*, and *R*. *mannitolilytica* (Table 1). Notably, none of the false positives strains Rs126, Rs129, K60, and UW349 detected by previous biovar 2 detection methods [13,22,25] were amplified by our RsSA1 and RsSA2 sets.

For the RsII qPCR primers and probe targeting *Rs* phylotype II strains, the forward primer RsII-F was designed by removing 2 nucleotides at the 5’ end and adding 4 additional ones at the 3’ end of Fegan and Prior’s standard PCR phylotype II-specific forward primer Nmult21:2F [6] (Table 2). The reverse primer RsII-R was designed to shorten the amplicon from 372- to 117-bp for qPCR (Table 2). When tested by uniplex qPCR, the RsII set recognized all 52 tested phylotype II strains, including the 34 IIB-1&2 strains, but none of the 38 phylotypes I, III and IV strains of *Rs*. It did not recognize the out-group bacteria *E*. *cloacae*, *X*. *campestris pv*. *campestris*, *P*. *syringae pv*. *syringae*, *X*. *citri*, *C*. *necator*, or *R*. *insidiosa*, although it recorded a high Cq reading of 38.20 for *R*. *pickettii* and did recognize *R*. *mannitolilytica* with a Cq of 20.79 (Table 1).

To create an effective internal control for detecting *Rs* in plant extracts by qPCR, the targeted region of the cytochrome oxidase subunit 1 (*cox1*) gene we previously used [23] was shortened from 641- to 144-bp, a length amenable to qPCR. This was achieved by keeping the reverse primer Cox1-R the same as before and redesigning the forward primer Cox1-qF (Table 2). When the cox1 primer pair Cox1-qF/R was tested in a standard PCR assay, it amplified a 144-bp PCR product from DNA templates extracted from 8 plant species including potato, tomato, tobacco, impatiens, salvia, vinca, oleander, and geranium (Table 3). When the probe Cox1-P-S, designed to recognize solanaceous plants, was tested together with the cox1 primers in uniplex qPCR against DNA extracted from the above 8 plant species, the probe recognized everything except geranium (Table 3). The probe Cox1-P-P, designed to recognize *Pelargonium*, amplified DNA only from geranium and not from any other tested plants (Table 3). When the two probes were tested together with the Cox1 primers (Cox1 duplexed probe set), the duplexed probe set recognized all 8 tested plant species (Table 3). When tested against the *Rs* strains and out-group bacteria in a uniplex assay, neither of the two Cox 1 probe sets amplified any of the tested bacteria (Table 1). For comparison, the Cox primers and probe set designed by Weller et al. [13] was tested against one potato, one tomato and five geranium DNA samples. Weller’s Cox set only detected DNA from the potato and tomato plants and not from any of the five geranium samples. Our Cox1 duplexed probe set, however, recognized not only the potato and tomato samples, but also the same 5 geranium DNA samples with mean Cq values of 21.66, 21.54, 22.16, 21.71, and 21.33.

**Table 3:** Test of our Cox1 primers and probes on different plant species by PCR and qPCR assays.

	Cox1 set		Potato^a^	Salvia	Tobacco	Oleander	Tomato	Impatiens	Vinca	Geranium
Test	Primer	Probe								
PCR	qF and R		+	+	+	+	+	+	+	+
	qF and R	P-S	+	+	+	+	+	+	+	-
qPCR	qF and R	P-P	-	-	-	-	-	-	-	+
	qF and R	P-S and P-P	+	+	+	+	+	+	+	+

^a^Total DNA from each of the tested plant species was extracted as previously described [23]. The presence or absence of PCR product or qPCR signal is indicated by + and-, respectively.

### qPCR amplification efficiencies and detection limits

**Fig 1:**
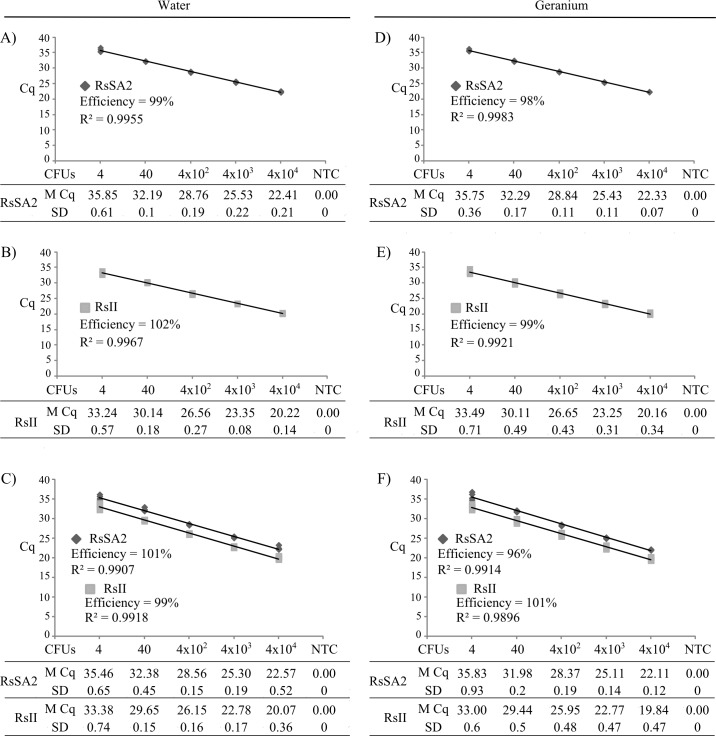
Comparison of efficiency and sensitivity for RsSA2 (A, C, D, F) and RsII (B, C, E, F) sets under both uniplex (A, B, D, E) and multiplex qPCR (C, F) conditions with 10-fold serial dilutions of *Ralstonia solanacearum* strain UW551 cells diluted in either sterile water or Qiagen-purified healthy geranium extracts. Mean (M) Cq value and standard deviation (SD) for each bacterial colony forming unit (CFU) was calculated from 4 replicates in two separate experiments. NTC: no template water control.

The standard curve of each uniplex and multiplex qPCR assay was determined using 10-fold serial dilutions of bacterial cells of the IIB-1 strain UW551 at concentrations of 4 to 4 x 10^4^ CFU per reaction in either sterile water (Fig 1A–1C) or in total plant DNA extracted from healthy geranium plants to mimic extracts of infected geranium plants (Fig 1D and 1E). When serial dilutions were made in water, the efficiencies of RsSA2 and RsII were 99% and 102%, respectively, under uniplex conditions (Fig 1A and 1B). When RsSA2, RsII and Cox1 were multiplexed together, the efficiencies were 101% and 99% for RsSA2 and RsII, respectively, similar to their efficiencies under uniplex conditions (Fig 1C). Similar efficiencies were obtained when purified healthy geranium DNA was used to dilute bacterial cell templates for qPCR (Fig 1D–1F). Although RsSA1 had an efficiency of 98% in uniplex assays, under multiplex conditions it increased to 155%, above the acceptable level of amplification efficiencies, and was thus not further studied in a multiplex qPCR assay. Cox1 efficiencies were confirmed to be acceptable under uniplex (101%) and multiplex (89%) conditions using serially diluted Qiagen-purified total DNA from geranium infected by UW551 (data not shown).

The limit of detection under uniplex and multiplex conditions was determined to be 4 CFU (2 μl of a 2 x 10^3^ CFU/mL sample) per reaction, when the template was diluted in either water or healthy geranium DNA (Fig 1). Detection of fewer than 4 CFU per reaction was not reliable, which is in agreement with previous studies [32]. Under uniplex and multiplex conditions, the Cq value for 4 CFU per reaction was between 35.46 and 35.85 for RsSA2, and between 33.00 and 33.49 Cq for RsII (Fig 1). Therefore, any Cq value above 36 cycles for RsSA2 and 34 cycles for RsII in our multiplex assay was deemed a negative result.

### Testing of bacterial strains and infected plant samples by multiplex qPCR assay

We developed a multiplex qPCR assay by combining the RsII, RsSA2, and Cox1 duplexed probe sets. We tested the multiplex qPCR assay in vitro against DNA extracted from our bacterial strain library. All the 34 IIB-1&2 strains were recognized by the RsSA2 set with Cq values ranging from 19.77 to 22.97, and no Cq values were obtained for the 65 non-IIB-1&2 or non-*Rs* strains (Table 1). Similarly, all 52 phylotype II strains were recognized by the RsII set with Cq values ranging from 17.17 to 21.99 and no Cq values were observed for the 38 non-phylotype II or non-*Rs* strains, except *R*. *mannitolilytica* (Cq of 22.51), *R*. *insidiosa* (Cq of 37.00), and *R*. *pickettii* (Cq of 34.71), though both *R*. *insidiosa* and *R*. *pickettii* were considered negative based on our cut off Cq value of 34 for RsII. The Cox1 duplexed probe set did not recognize any of the bacterial strains (Table 1).

We also tested the multiplex qPCR assay in planta against Qiagen-purified DNA extracted from 83 plants, including 18 water control plants and 65 symptomatic (wilted) tomato, geranium, tobacco and potato plants (Table 4). In all 83 cases, our Cox1 duplexed probe set detected the plant samples with a mean Cq value of 20.66 (Table 4). Our RsII set detected all 60 wilted plant samples infected by the phylotype II strains UW551, UW344, K60, Rs5, or UW349 with a mean Cq value of 17.31 (Table 4), but neither the 5 plants infected with the phylotype I strain GMI1000 nor the 18 water control plants gave a positive reaction. Among the 60 phylotype II infected plants, our RsSA2 set detected the 34 plants infected by the IIB-1 strains UW551 or UW344 with a mean Cq value of 17.89 (Table 4).

**Table 4:** Test of symptomatic plants artificially infected with *Ralstonia solanacearum* strains by our multiplex qPCR assay.

			# of plants artificially infected with:			
Plant		# of plants	Water	Phylotype II		Non-phylotype
				IIB-1 strain^a^	Non-IIB-1&2 strain^b^	II strains^c^
Tomato		29	4	14	6	5
Geranium		23	3	9	11	0
Tobacco		5	3	NT^d^	2	0
Potato		26	8	11	7	0
Total # of plants		83	18	34	26	5
Test	Name of set	Mean Cq ± SE^e^	Cq ± SE			
	Cox1 duplexed probe	20.66 ± 0.15	19.75 ±0.30	20.77 ±0.20	21.09 ±0.26	21.05 ±0.91
Multiplex qPCR	RsII	17.31 ± 0.22	-	16.84 ±0.32	17.88 ±0.25	-
	RsSA2	17.89 ± 0.31	-	17.89 ± 0.31	-	-

^a^Strain UW551 or UW344.

^b^Strain K60, UW349, or Rs5.

^c^Strain GMI1000.

^d^Not tested.

^e^Mean Cq was calculated from a total of 83 tested plant samples for Cox1 duplexed probe set including 18 water control and 5 non-phylotype II strain-infected plants, 60 phylotype II-infected plant samples for RsII set, 34 IIB-1 strain-infected plant samples for RsSA2 set, respectively. SE: standard error.

**Fig 2:**
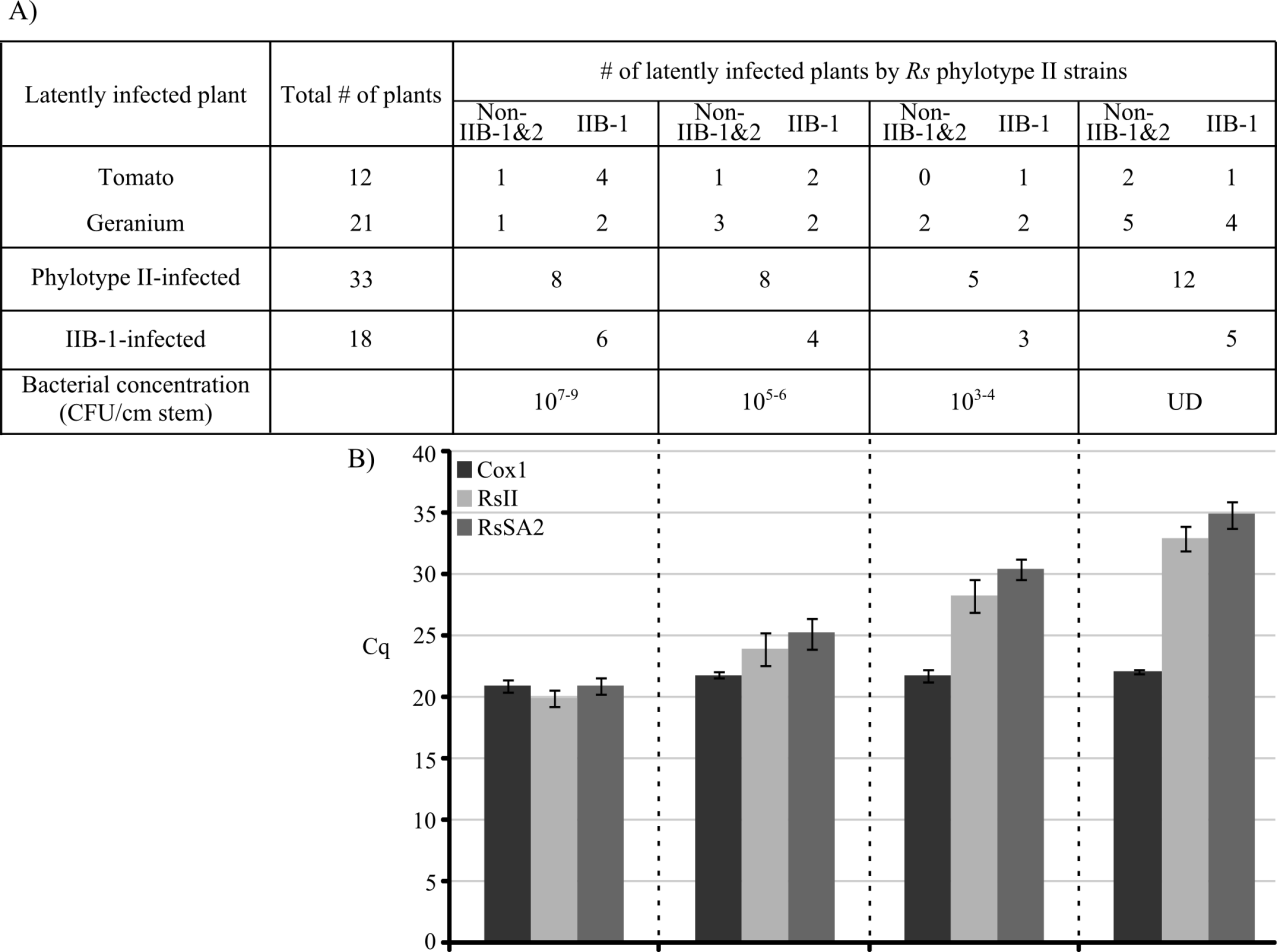
Test of latently infected plants by phylotype II IIB-1 or non-IIB-1&2 strains of *Ralstonia solanacearum* by dilution plating (A) and qPCR (B). Every sample had a positive Cox1 signal and the mean Cq was 21.72 (±0.17, n = 33). Asymptomatic plants with 10^7−9^, 10^5−6^, and 10^3−4^ CFU/ml/cm of *Rs* had a mean Cq of 19.91 (±0.68, n = 8), 23.97 (±1.27, n = 8), and 28.28 (±1.31, n = 5) for RsII and 20.93 (±0.67, n = 6), 25.21 (±1.23, n = 4), and 30.42 (±0.8, n = 3) for RsSA2, respectively. Asymptomatic plants with undetectable amounts of *Rs* (UD) had average Cq values of 32.97 (±0.93, n = 12) for RsII and 34.9 (±1.09, n = 5) for RsSA2. Error bars represent standard error.

We further tested our multiplex qPCR assay with Qiagen-purified DNA extracted from a total of 86 asymptomatic tomato (32) and geranium (54). Similar to the symptomatic plants, our Cox1 duplexed probe set detected all 86 asymptomatic plants with a mean Cq value of 22.37 and standard error (SE) of 0.18. Of these 86 asymptomatic plants, 33 (12 tomatoes and 21 geraniums) were identified as latently infected by *Rs* with our multiplex qPCR assay, and were further tested by dilution plating to determine if they contained detectable amounts of *Rs* cells (Fig 2A). Twenty-one of the 33 plants were found to contain 10^3^ to 10^9^ CFU/ml/cm stem, while no bacterial cells were isolated from 12 other inoculated plants (Fig 2A). The qPCR detection was observed in a bacterial concentration dependent manner when Cqs were averaged for each detected cell concentration range, with values ranging from 28.28 (SE = 1.31) (10^3−4^ CFU/ml/cm stem) to 19.91 (SE = 0.68) (10^7−9^ CFU/ml/cm stem) for the RsII set (Fig 2B). A similar concentration-dependent manner was observed for the RsSA2 set when detecting the 18 plants infected by the IIB-1 strains, with Cq values ranging from 30.42 (SE = 0.8) for 10^3−4^ to 20.93 (SE = 0.67) for 10 ^7–9^ CFU/ml/cm stem (Fig 2B). Even for the 12 plants with undetectable amounts of bacterial cells, our RsII set recognized those plants as infected with an average Cq value of 32.97 (SE = 0.93) (Fig 2B). Similarly, our RsSA2 set recognized the five of the twelve plants with undetectable amount of bacterial cells that had been inoculated with IIB-1 strains, with an average Cq value of 34.90 (SE = 1.09) (Fig 2B).

### Extraction buffer for a quick extraction of geranium samples for qPCR analysis

The optimum concentration and combination of our extraction buffer were determined to be 100 mM Tris-HCl, 150 mM NaCl, 1 M Betaine monohydrate, 10% polyvinyl-pyrrolidone, and 5% Triton X-100, pH 9.3 for a quick, easy and less costly extraction before a qPCR assay. Extraction using our buffer requires minimum sample processing that takes approximately 15 min to complete. Based on our experience and the literature [25], geranium samples were more and tomato samples were less prone to causing inhibition during PCR and qPCR when compared to potato samples. We therefore used geranium as the standard to test our extraction method for its effectiveness in removing or inactivating qPCR inhibitors before qPCR assays (Table 5). When compared to Qiagen’s DNeasy Plant Mini Kit and Epicentre’s QuickExtract Seed DNA Extraction Solution, as well as extraction with water as a control, our extraction method coupled with our multiplex qPCR assay detected down to 40 CFU per reaction, while extraction with water yielded no qPCR signal (Table 5A). Epicentre’s extraction method yielded no qPCR signals for all of the 5 bacterial concentrations tested ranging from 4 to 10^4^ CFU per reaction (Table 5A). Qiagen’s extraction method allowed detection of 4 CFU per reaction (Table 5A), although the extraction took almost two hours to complete. Our extraction buffer has a 2-hour optimum effective time if plant samples were added and stored at room temperature, since qPCR inhibition was observed between 2–4 hours in some cases (data not shown). The effective time of our buffer can be prolonged for a few hours to at least a day if geranium samples were added to the extraction buffer on ice and stored either at 4°C or at -20°C. It did not seem to matter if the step of 10 minutes at 65°C was performed before or after the extracted samples were frozen.

**Table 5:** A simultaneous comparison of extraction methods followed by our multiplex qPCR assay.

A. Detection limit using healthy geranium DNA extracted by different extraction methods and spiked with known numbers of bacterial cells of *Rs* strain UW551													
		Extraction method											
Bacterial cell		Qiagen			This study			Epicentre			Water		
concentration (CFU)		RsSA2	PhyII	Cox1	RsSA2	PhyII	Cox1	RsSA2	PhyII	Cox1	RsSA2	PhyII	Cox1
4 x 10^4^		+	+	+	+	+	+	-	-	-	-	-	-
4 x 10^3^		+	+	+	+	+	+	-	-	-	-	-	-
4 x 10^2^		+	+	+	+	+	+	-	-	-	-	-	-
40		+	+	+	+	+	+	-	-	-	-	-	-
4		+	+	+	-	-	-	-	-	-	-	-	-
B. qPCR results using infected symptomatic geranium plants													
Strain	plant												
	1	-	16.82	21.22	-	20.14	26.34	-	-	-	-	-	-
	2	-	16.73	23.08	-	21.95	30.45	-	-	-	-	-	-
K60 or	3	-	24.59	21.93	-	31.52	29.71	-	-	-	-	-	-
Rs5	4	-	23.02	22.78	-	26.98	30.93	-	-	-	-	-	-
	5	-	28.48	22.29	-	33.49	30.12	-	-	-	-	-	-
	6	-	20.76	22.50	-	26.14	30.96	-	-	-	-	-	-
	1	19.12	18.28	22.16	22.47	22.48	27.02	-	-	-	-	-	-
	2	18.62	16.97	21.53	22.61	22.07	26.82	-	-	-	-	-	-
UW551	3	19.92	18.38	19.79	23.01	21.37	24.25	-	-	-	-	-	-
or	4	19.58	17.90	20.60	22.75	21.37	29.84	-	-	-	-	-	-
UW344	5	19.12	17.66	20.73	24.54	22.54	26.93	-	-	-	-	-	-
	6	17.94	16.03	20.93	22.88	21.17	24.80	-	-	-	-	-	-
C. qPCR results using infected asymptomatic geranium plants													
Strain	plant												
	1	-	20.99	21.38	-	26.5	25.99	-	-	-	-	-	-
K60 or	2	-	32.93	25.54	-	-	30.11	-	-	-	-	-	-
Rs5	3	-	23.63	24.14	-	28.83	29.77	-	-	-	-	-	-
	4	-	23.57	26.27	-	27.04	30.71	-	-	-	-	-	-
	5	-	30.51	25.46	-	-	31.46	-	-	-	-	-	-
	6	-	22.07	22.03	-	28.20	30.39	-	-	-	-	-	-
	1	29.27	27.54	21.81	23.61	22.53	26.17	-	-	-	-	-	-
	2	30.76	28.75	21.65	34.07	33.46	27.33	-	-	-	-	-	-
UW551	3	24.90	23.93	23.52	35.80	33.93	28.27	-	-	-	-	-	-
or	4	22.13	20.36	22.64	29.90	28.33	26.61	-	-	-	-	-	-
UW344	5	24.69	23.17	25.49	31.46	29.95	28.81	-	-	-	-	-	-
	6	22.23	20.65	24.45	30.07	27.44	30.54	-	-	-	-	-	-
	1	-	-	22.24	-	-	26.17	-	-	-	-	-	-
	2	-	-	22.88	-	-	27.49	-	-	-	-	-	-
Water	3	-	-	22.16	-	-	27.15	-	-	-	-	-	-
	4	-	-	22.58	-	-	27.35	-	-	-	-	-	-
	5	-	-	22.93	-	-	27.01	-	-	-	-	-	-
	6	-	-	24.20	-	-	29.10	-	-	-	-	-	-
D. qPCR results using infected symptomatic tomato plants													
	1	19.52	18.42	19.35	21.08	20.12	26.11	19.65	18.96	24.61	26.75	26.24	27.09
UW551	2	19.53	18.31	18.66	23.17	22.01	24.56	22.97	21.96	26.31	27.71	26.83	27.78
	3	19.26	18.11	19.60	22.46	20.92	25.42	20.71	19.95	24.31	26.48	25.61	27.64
	4	20.21	19.77	19.12	23.67	23.24	25.32	22.26	22.24	24.11	26.16	26.24	26.13

We also tested our extraction method using symptomatic and asymptomatic geranium plants artificially infected with the IIB-1 strains, or non-IIB-1&2 phylotype II strains. All of the geranium plants infected by IIB-1 strains, whether wilted or asymptomatic, were detected by our multiplex qPCR when the extraction was done using either our extraction method or Qiagen’s, but none were detected if extracted using Epicentre’s method or water (Table 5B and 5C). For geranium plants infected with a non-IIB-1&2 strain, all but two of the 12 samples were detected by our extraction method, which yielded high Cq readings of 32.93 and 30.51 by using Qiagen’s extraction method, suggesting that these two latently infected samples may contain fewer than 40 CFU per reaction (Table 5B and 5C). The Cq ranges detected by our RsSA2, RsII, and Cox1 sets from the same tissue samples were 22.47 to 35.80, 20.14 to 33.93 and 24.25 to 31.46 for infected geranium DNA extracted with our extraction method, respectively, as compared to 17.94 to 30.76, 16.03 to 32.93 and 19.79 to 26.27 with Qiagen’s extraction method (Table 5B and 5C). No qPCR signals were detected for the infected geranium samples extracted using Epicentre’s method or water (Table 5B and 5C). We also simultaneously extracted DNA from four symptomatic tomato samples infected with UW551 strain for qPCR analysis and found that all four techniques were able to provide detection (Table 5D). DNA extraction with our buffer is more sensitive than extraction with water alone (RsSA2 and Rs II, P<0.01 and Cox1, P<0.05) and is similar to extraction with Epicentre’s method for tomato plant samples.

## Discussion

We developed a multiplex qPCR assay that simultaneously identifies and confirms *Rs* IIB-1&2 strains, and includes an internal plant control that recognizes many plant species including the *Rs* hosts such as potato, tomato, tobacco and geranium. This is a significant improvement over previous qPCR detection methods for multiple reasons. First, the current qPCR assay is a duplex assay that detects the IIB-1&2 strains and either *Rs* species complex strains or an internal plant control, but not all three. Our assay is the only one combining detection of the IIB-1&2 strains at two different levels (phylotype and sequevar) and an internal plant control into one multiplex, saving time, money and labor and also increasing the confidence of the assay. Second, our primers and probe sets for the IIB-1&2 strains target non-phage related stable regions of the chromosome and eliminated false positives associated with previous qPCR assays, improving the specificity and reliability of the detection. Third, none of the previous multiplex qPCR assays has internal plant primers and probes that can recognize geranium, an important host of the IIB-1&2 strains. Our Cox1 duplexed probe set recognized all major hosts of *Rs* including tomato, potato, tobacco and geranium, improving the robustness of the internal plant control in the multiplex qPCR assay.

We designed two sets of primers and a probe for specific detection of the IIB-1&2 strains of *Rs* by targeting two different regions of the bacterial chromosomal DNA. One region (RsSA1) was previously used in our multiplex PCR assay, and the other (RsSA2) was identified independently by our group and two other groups as being IIB-1&2-specific [20,22,23]. Neither is located in predicted phage regions [23], nor is RsSA2 located in an alternative codon usage region [20], suggesting they are stable targets. The RsSA1 and RsSA2 sets did not recognize any of the previously identified false positives including Rs126, Rs129, K60 and UW349 [23,27]. Strain UW349, in particular, was used previously as a representative of IIB-1&2 strains and gave strong false positive results with previous assays [22,25]. Although for unknown reasons RsSA1 could not be multiplexed with the RsII and Cox1 sets, it can still be used in a uniplex qPCR as an independent assay for the IIB-1&2 strains, since its target is spaced a considerable distance from the RsSA2 target region.

The recent proposed nomenclature change to the *Rs* species complex heightened our interest in designing primers and a probe to the *Rs* phylotype II strains to add another layer of detection for IIB-1&2 strains [7]. Under the proposed change, only the current phylotype II strains would continue to be *Rs*. Our phylotype II primers and probe correctly identified all phylotype II strains in our collection, although *R*. *mannitolilytica*, an opportunistic human pathogen, was also recognized. The other two opportunistic human pathogens, *R*. *insidiosa* and *R*. *pickettii*, had high Cq values for our RsII set, but the readings are above the cut-off value for being considered positive as *Rs* phylotype II strains. We included these opportunistic human pathogens out of extreme precaution for our testing, since these three *Ralstonia* species are the most closely-related to the *Rs* species complex [33]. We attempted to re-design the phylotype II primers in the 16S-23S intergenic region to eliminate the signal for *R*. *mannitolilytica*, but for unknown reasons the new primers resulted in no amplification of some of the phylotype II strains in our collection (data not shown). Attempts to design primers based on other regions of the genome identified as phylotype II specific through genome comparisons yielded similar results (data not shown). Since *R*. *mannitolilytica* is a human pathogen and not a plant or soil-associated pathogen like *Rs*, the mistarget should not interfere with the usefulness of the assay, especially given that the purpose of RsII is to confirm IIB-1&2 detection, rather than as a stand-alone method for detection of phylotype II strains.

We previously designed a Cox1 primer pair that recognizes 8 different plant species including tomato, potato, tobacco, impatiens, salvia, vinca, oleander, and geranium [23]. Shortening the amplicon at the 5’ end for qPCR purposes did not affect the qPCR primers’ specificity by a standard PCR assay or with SYBR Green in a qPCR assay, although two probes had to be designed and used in order to recognize DNA from all 8 diverse plant species in a TaqMan qPCR assay. The two probes can be used individually or duplexed together in either a uniplex or multiplex qPCR assay, depending on the target plant samples.

We tested the multiplex qPCR assay against both symptomatic and asymptomatic plants to ensure that the assay works in planta. These samples were obtained through artificial infection in our greenhouse, since it is very difficult to obtain plant samples naturally infected by the IIB-1&2 strains due to the strict regulations on SA pathogens. From latently infected plants, we were only able to isolate target bacterial colonies by dilution plating when the plants contained at least 10^3^ CFU/ml/cm stem, which is consistent with the limit of detection found by other groups for dilution plating [29]. Our multiplex qPCR assay, however, can detect as few as four CFU of *Rs* per reaction in spiked Qiagen-extracted healthy geranium DNA samples. It also detected target *Rs* strains in both symptomatic and asymptomatic plants, even when some of the asymptomatic plant samples contained undetectable amounts of bacterial cells by dilution plating.

A method of quick extraction is desirable when processing a high volume of samples. The challenge for DNA detection following quick extraction is the presence of PCR inhibitors in the plant samples, especially those from geranium plants [25]. Previous comparisons showed that commercial DNA extraction kits like Qiagen’s gave the most reproducible extraction results, but they were laborious and the most expensive [29]. Other published methods, such as magnetic capture hybridization and immunomagnetic separation methods, require significant processing time and specialized and costly materials [25]. Our extraction method using the extraction buffer we developed requires little sample processing time (approximately 15 minutes) and effort. The stem tissue/bacterial cell is lysed by the 5% triton x-100 under high pH conditions [34], and heating at 65°C with gentle agitation. The PVP in the buffer is required to combat PCR inhibition [35], and the betaine is necessary to prolong the usability of the sample [36]; without betaine, PCR inhibition occurred within 2 hours of the sample entering the buffer (data not shown). Compared to the commercial QuickExtract Seed DNA Extraction Solution by Epicentre, our method was as easy to use and had a similar sensitivity in detecting infected tomato samples. It also has the advantages of being cheaper to obtain and being able to detect both symptomatic and asymptomatic geranium samples, while the Epicentre method was unable to detect any of the geranium samples. Compared to Qiagen’s DNeasy Plant Mini Kit, our extraction was about 10 times less sensitive; however, it was easier, quicker and less costly to use for processing a large number of plant samples. The reduced sensitivity of our buffer compared to Qiagen’s was most evident in two asymptomatic, non-IIB-1&2 infected geranium samples that tested positive using Qiagen’s method but not by ours, suggesting that our extraction method is not suitable for extraction of plant samples with bacterial cell level below its detection limit of 40 cells per reaction, or 4 x 10^4^ CFU/mL/cm stem.

We have successfully designed qPCR primers and probes to target phylotype II strains and IIB-1&2 strains of *Rs*, as well as a diverse group of plant species including tomato, potato, tobacco, and especially geranium. Our multiple qPCR assay can be used as either an initial test of suspected *Rs*-infected samples or for further detection/identification of IIB-1&2 strains for an ELISA- or immunostrip-positive sample. A sample should be considered IIB-1&2 positive only when recognized by both RsSA2 and RsII sets with similar Cq values (within 2–3 cycles). The identity of IIB-1&2 can be further confirmed using the uniplex RsSA1 set, since it targets a different stable gene region on the *Rs* genome. If the strain is isolated, its biovar nature can be determined by the improved biovar test [37]. Since the RsII set is designed as a confirmation of the phylotype II identity of the IIB-1&2 strains, not as an independent test for phylotype II strains, it needs to be used together with the RsSA2 set. If a sample is tested positive by RsII but negative by RsSA2, it is unlikely that it is a IIB-1&2 strain. Whether it is a *Rs* strain, however, requires additional testing using *Rs*-species complex-specific primers such as RsSC [23] or 759/760 [14] to exclude the possibility that it is the non-*Rs* strain *R*. *mannitolilytica*, although the two *Ralstonia* species occupy very different ecological niches (plants vs human). A plant sample should test positive for Cox1 regardless of whether it contains *Rs*, otherwise the result is not reliable due to either qPCR inhibition or an unsuccessful DNA extraction from plant samples. In the U. S., any sample that tests positive for *Rs* is subject to select agent regulations unless meeting criteria for exclusion [31]. Coupled with the quick extraction method we developed, our assay can be used as a quick, easy, and reliable method for specific detection and differentiation of the IIB-1&2 strains of *Rs*.
